# Alterations in submaximal cardiopulmonary indicators in CVD patients participating in APBCRE—a comprehensive CPET-based study

**DOI:** 10.3389/fcvm.2026.1825961

**Published:** 2026-07-07

**Authors:** Yukun Liang, Mei Ma

**Affiliations:** 1 School of Medical Technology, Tianjin University of Traditional Chinese Medicine, Tianjin, China; 2 Department of Rehabilitation Medicine, Tianjin Chest Hospital, Tianjin, China

**Keywords:** adaptive postural balance cardiac rehabilitation, cardiorespiratory fitness, cardiovascular disease, exercise rehabilitation, oxygen uptake efficiency slope

## Abstract

**Objective:**

This study aims to evaluate the clinical efficacy of a one-month Adaptive Postural Balance Cardiac Rehabilitation (APBCRE) program for patients with cardiovascular disease (CVD) using cardiopulmonary exercise testing (CPET), and to assess the validity of the oxygen uptake efficiency slope (OUES) as a measure of cardiopulmonary rehabilitation outcomes.

**Design:**

The study implemented a one-month exercise rehabilitation program consisting of 12 sessions, held three times a week for 50–70 min each. All sessions were conducted on a one-on-one basis, with each patient receiving direct supervision throughout the program from certified cardiac rehabilitation nurses and physical therapists. A CPET test was administered by the same therapist before and after the program, and exercise instruction was provided prior to each test.

**Result:**

Participants were stratified into two age groups for analysis. After completing the rehabilitation program, significant improvements were observed in WAT (56.86 ± 25.43 to 71.93 ± 26.64 w, *p* = 0.0009), VE at AT (29.64 ± 7.91 to 35.38 ± 12.22 mL/min/kg, *p* = 0.006), VO_2_ at AT (11.74 ± 3.20 to 13.79 ± 3.48 mL/min/kg, *p* = 0.002) and OUES [1,485.20 ± 377.54 to 1,610.60 ± 419.81 mL/(min·L), *p* = 0.035]. The change in VO_2_ at AT was more pronounced than in VE at AT. The primary evaluation metrics were VO_2_ at AT and OUES, while secondary metrics included RER, HR, VE at AT, and VE/VCO_2_.

**Conclusion:**

The findings suggest that APBCRE is a potentially effective rehabilitation method for patients with cardiovascular disease, leading to significant improvements in exercise tolerance within one month. Furthermore, the OUES was found to be a potentially valuable indicator for assessing changes in cardiorespiratory fitness among young individuals during sports rehabilitation.

## Introduction

Cardiovascular disease (CVD) is the leading cause of death and disability worldwide (1). A large cohort study demonstrated that changes in cardiopulmonary exercise capacity over time reflect corresponding changes in mortality risk, independent of other comorbidities (2). Furthermore, increased physical activity levels are associated with a reduced incidence of cardiovascular disease (3). Given these findings, developing appropriate exercise prescriptions and guidance for patients is crucial. Cardiopulmonary exercise testing (CPET) is considered the gold standard for assessing cardiopulmonary fitness. Traditionally, exercise prescriptions for patients with cardiovascular disease and assessments of exercise tolerance have typically relied on peak oxygen consumption (VO_2_peak), maximum heart rate (HRmax), and anaerobic threshold (AT) measured via cardiopulmonary exercise testing (CPET) (4–6). Although VO_2_peak measurement is considered effective for patients with cardiovascular disease (7), factors such as patient anxiety, motivation, individual differences in tolerance, and lack of exercise experience may lead to premature termination of exercise, thereby affecting the accuracy of results (8). Maximum heart rate is similarly influenced by these factors and medication (9). Furthermore, in clinical practice, many patients experience significant fatigue as they approach their anaerobic threshold and often choose to terminate the exercise early, which further undermines the reliability of the measurements.

To address these limitations, several methodological improvements have been proposed, including testing during a secondary validation phase (10), criteria for determining the plateau during true VO_2_peak testing (11), age-based correction formulas for predicting VO_2_peak (12), and RPE-guided protocols (RPE, Ratings of Perceived Exertion) (13). Although these methods can improve measurement validity, they require additional testing time, advanced equipment, and the patient's willingness to repeat exercise at near-maximal intensity—which is often difficult to achieve in routine outpatient cardiac rehabilitation, particularly for elderly or high-risk patients. Therefore, the submaximal intensity index oxygen uptake efficiency slope (OUES), which is unaffected by the patient's exercise intensity, requires only a single test, and yields reproducible results, will be the focus of our study. The formula for OUES is *VO*_2_ = *a* × *lgVE* + *b* (a is OUES).

An early exploratory study suggested that the OUES may be useful for assessing exercise performance in heart failure patients who are unable to undergo maximal exercise testing (14). Subsequently, a study involving 425 patients with cardiovascular disease showed that after 3 months of physical training, participants' peak oxygen consumption and OUES increased significantly, and the two variables were well correlated. This indicates that the secondary outcome measure OUES has clinical utility in quantifying exercise performance and is sensitive to training in patients with cardiovascular disease (15). Similarly, the correlation between OUES and peak oxygen consumption was also well-established in patients with epilepsy (16). Furthermore, the results of a study examining OUES values at different stages demonstrated that OUES values measured during aerobic exercise phases serve as a valid surrogate marker for maximal exercise testing (17). This conclusion was also confirmed in endurance athletes (18).

Adaptive Postural Balance Cardiac Rehabilitation (APBCRE) is a novel training method that has shown promise in improving exercise tolerance in patients with CVD. Unlike traditional single-modality training, APBCRE combines breathing exercises with warm-ups, aerobic exercise, resistance training, and flexibility training (19).

Although a growing body of evidence supports OUES as an alternative to submaximal-intensity exercise, three knowledge gaps remain. First, most validation studies of OUES have been conducted using traditional aerobic protocols in populations with heart failure or following revascularization; its responsiveness to multimodal rehabilitation programs that integrate balance, respiratory, and resistance training elements has not yet been systematically investigated. Second, Second, it remains unclear whether OUES demonstrates evaluative value for CVD patients of different ages in short-term (≤1 month) cardiac rehabilitation. Third, APBCRE—a novel program combining adaptive postural balance training with traditional aerobic and resistance training—has shown promise in cardiac rehabilitation, but its effects on submaximal cardiopulmonary parameters have not yet been quantified. To address these gaps, this study set two objectives: (1) to evaluate the short-term (1-month) effects of APBCRE on submaximal CPET parameters in CVD patients; (2) To evaluate whether age-stratified OUES can serve as an effective submaximal indicator of rehabilitation-induced changes in cardiopulmonary fitness.

## Materials and methods

**Figure 1:**
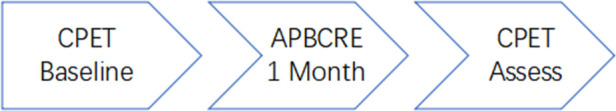
Flowchart of this study. CPET cardiopulmonary exercise test, RMR resting metabolic rate, APBCRE adaptive postural balance cardiac rehabilitation exercise.

A total of forty-two outpatients with cardiovascular diseases from Tianjin Chest Hospital were included in the study, and demographic characteristics were recorded. All participants were independently assessed by the same researcher using standardised criteria and received standardised drug treatment. This study is an exploratory single-arm study; no prior sample size calculation was performed, and further validation studies are required to confirm the findings. This study was registered at the Chinese Clinical Trial Registry (ChiCTR) under the registration number ChiCTR2300078066. This study received funding from the Tianjin Key Medical Specialties (Profession) Construction Project TJYXZDXK-055B and Tianjin Science and Technology Program Project TJWJ2022ZD007. The study protocol was approved by the local ethics committee. All subjects signed an informed consent form before enrollment. As shown in Figure 1, the study design comprised a one-month exercise rehabilitation program with clinical assessments conducted before and after the intervention. The one-month rehabilitation program consisted of 12 sessions three times per week.

Age stratification at 55 years was pre-specified based on (i) the well-established acceleration of age-related decline in skeletal muscle oxidative capacity, ventilatory reserve, and arterial compliance beyond the fifth decade (20), and (ii) the median age commonly reported in Chinese CVD cardiac-rehabilitation cohorts (21). Subgroup analyses were conducted to explore whether the responsiveness of OUES to APBCRE differed between younger (<55 years) and older (≥55 years) patients. Results from subgroup analyses are interpreted as exploratory and hypothesis-generating.

The individualized ramp rate for CPET was determined using the Wasserman formula (22) to target a symptom-limited exercise duration of 8–10 min. Unloaded oxygen uptake (VO_2,unloaded_, mL/min) was estimated as:

$$VO_{2,\mathrm{unloaded}}=150+6\times W_{\mathrm{measured}}$$

where W_measured_ is measured body weight (kg).

Sex-specific parameters were then derived. Sex-specific parameters are calculated as follows:

For males:

$$\begin{array}{rl} & k_{\mathrm{male}}=50.72-0.372\times \mathrm{age},(\mathrm{age}\;\mathrm{in}\;\mathrm{years})\\ & W_{\mathrm{ideal},\;\mathrm{male}}=0.79\times \mathrm{height}-60.7,(\mathrm{height}\;\mathrm{in}\;\mathrm{cm}) \end{array}$$

For females:

$$\begin{array}{rl} & k_{\mathrm{female}}=22.78-0.17\times \mathrm{age}\\ & W_{\mathrm{ideal},\mathrm{female}}=0.65\times \mathrm{height}-42.8 \end{array}$$

Here, k is the cycling coefficient (implied units: mL·kg^−1^·min^−1^) and Wideal is ideal body weight (kg).

The predicted peak oxygen uptake (VO_2,peak_, in mL/min) is determined by comparing measured body weight (W_measured_) with ideal body weight (W_ideal_):

$$VO_{2,\mathrm{peak}}=\{\begin{array}{c} (W_{\mathrm{ideal}}+W_{\mathrm{measured}})/2\times k),W_{\mathrm{measured}}<W_{\mathrm{ideal}}\\ (W_{\mathrm{measured}}+43)\times k,W_{\mathrm{measured}}=W_{\mathrm{ideal}}\\ W_{\mathrm{ideal}}\times k+6\times (W_{\mathrm{measured}}-W_{\mathrm{ideal}}),W_{\mathrm{measured}}>W_{\mathrm{ideal}} \end{array}$$

The constant 43 in the second case has units of kg (added to body weight), and the constant 6 has units of mL·kg^−1^·min^−1^ (multiplied by excess weight to contribute to oxygen uptake).

The theoretical ramp rate (W/min) was computed as:

$$\mathrm{Ramp}=\frac{VO_{2,\mathrm{peak}}-(150+6\times W_{\mathrm{measured}})}{100}$$

The denominator 100 is based on the classic relationship that each 1 W increase in work rate corresponds to an oxygen uptake increase of approximately 10 mL/min; thus, dividing *Δ*VO_2_ (in mL/min) by 100 gives the ramp rate in W/min.

In patients with cardiovascular disease, the theoretical value was reduced by approximately 4.84 W to prevent premature fatigue and ensure test feasibility, with further fine-tuning based on clinical status and medication use (22). The anaerobic threshold (AT) was determined non-invasively using the V-slope method via the CPET system. In clinical practice, the test system calculates ramp rate and AT based on the above methods.

The primary outcomes were: (i) change in oxygen uptake at the anaerobic threshold (VO_2_ at AT, mL·min^−1^·kg^−1^) and (ii) change in OUES (*VO*_2_ = *a* × *lgVE* + *b*, a is OUES), both measured before and after the 1-month APBCRE program. Secondary outcomes included changes in heart rate at AT (HR at AT, bpm), respiratory exchange ratio at AT (RER at AT), minute ventilation at AT (VE at AT, L·min^−1^), the ventilatory equivalent for CO_2_ (VE/VCO_2_ slope), and work at AT.

### Inclusion and exclusion criteria

Inclusion criteria were: (1) age 18–75 years; (2) a confirmed diagnosis of cardiovascular disease, including coronary heart disease (CHD), prior myocardial infarction (MI), arrhythmia, or valvular heart disease; (3) for patients who had undergone percutaneous coronary intervention (PCI), at least one week must have elapsed since the procedure; (4) for patients who had undergone coronary artery bypass grafting (CABG), at least one month must have elapsed, in line with current cardiac rehabilitation guidelines; and (5) clinically stable on guideline-directed medical therapy prior to enrollment. Exclusion criteria were: contraindications to symptom-limited exercise testing as defined by AHA guidelines (23), including unstable hemodynamics, decompensated heart failure, unstable angina, acute MI within the preceding 7 days, severe uncorrected congenital heart disease, and severe musculoskeletal limitations preventing cycle ergometry.

### Exercise program

This training program is based on a published paper on Adaptive Postural Balance Cardiac Rehabilitation Exercise (APBCRE) (19), which includes breathing exercises and warm-up, aerobic, resistance and flexibility exercises. The first part usually lasts 5–15 min for any risk level. Part I focuses on improving coordination and balance and includes stretches for the upper body, legs, lower back, and other areas of the body. Part II is a moderate intensity endurance exercise. Intensity is controlled at 40%–60% of AT, 60%–70% of maximal heart rate, Borg rating of 12–13, and the duration of aerobic exercise is usually 30 min. the third part is resistance exercise using an exercise bike for 10–15 min. the resistance power of the bike is adjusted according to the risk level and the VO_2_ value during AT. the second part is to improve coordination and balance of the body. The fourth part of the program consists of 5–10 min of low-intensity aerobic training aimed at slowing down the return of blood from the skeletal muscles to the heart, which can be effective in preventing a significant increase in cardiac stress. In summary, the total exercise time per session is typically 50–70 min, varying with the patient's physical function. Patients need to complete 12 training sessions in a month. Critical steps in APBCRE balance training, as shown in Figure 1.

All training sessions were conducted on a one-to-one basis with a certified cardiac rehabilitation nurse and a physiotherapist directly supervising each patient throughout the session. Exercise intensity was individualized based on the patient's risk stratification (as shown in Table 1) and the AT-derived heart rate, VO_2_, and Borg ratings obtained at baseline CPET. Sessions took place in the cardiac rehabilitation unit of Tianjin Chest Hospital, equipped with continuous telemetry monitoring (ECG, SpO_2_, blood pressure) and emergency resuscitation facilities.

**Table 1:** AACVPR proposes 3 levels of exercise risk for CVD patients.

Items	Low risk	Moderate risk	High risk
Exercise Testing	No complex ventricular arrhythmias during exercise testing and recovery; No angina or other significant symptoms (such as abnormal respiratory patterns, dizziness, or syncope) during exercise testing and recovery; Normal hemodynamic responses during exercise testing and recovery (i.e., appropriate increases and decreases in heart rate and blood pressure with increasing workload); Exercise capacity ≥7 METs.	Angina or other significant symptoms (e.g., abnormal respiratory patterns, dizziness, or syncope) during high—intensity exercise (≥7 METs); Mild to moderate ischemia during exercise testing or recovery (ST—segment depression ≤2 mm);	Complex ventricular arrhythmias during exercise testing or recovery; Angina or other significant symptoms (e.g., abnormal respiratory patterns, dizziness, or syncope) during low—intensity exercise (<5 METs); Severe ischemia during exercise testing or recovery (ST—segment depression ≥2 mm);
Non -Exercise Testing	Resting left ventricular ejection fraction ≥50%; No history of myocardial infarction or revascularization; No complex ventricular arrhythmias at rest; No chronic heart failure; No symptoms or signs of ischemia after illness or surgery; No clinical depression.	Resting left ventricular ejection fraction 40%—49%; No clinical depression.	Resting left ventricular ejection fraction <40%; History of myocardial infarction or revascularization; Complex ventricular arrhythmias at rest; Chronic heart failure; Symptoms or signs of ischemia after illness or surgery; Clinical depression.
Note	Each item is met for low risk.	Not meeting high—risk or low—risk criteria is moderate risk.	The presence of any high—risk item is high risk.

Training parameters are based on CPET test results, with the subject's metrics maintained near the anaerobic threshold. The criteria for terminating the exercise are as follows the AHA guidelines (23, 24): compared to the anaerobic threshold (AT), a rise in systolic blood pressure (SBP) of ≥40 mmHg, a rise in diastolic blood pressure (DBP) of ≥20 mmHg, or an increase in heart rate of more than 30%; the onset of discomfort or symptoms such as dyspnea, dizziness, or chest pain; or the occurrence of acute cardiovascular events such as unstable angina, acute heart failure, or malignant arrhythmias. Until termination criteria are met, we use the patient's own exercise tolerance as the indicator for stopping training.

### Measurement methods and equipment

Cardiopulmonary exercise testing (CPET) was performed on an exercise cardiopulmonary function measurement system (Oxycon Mobile, JAEGER, Germany) (CPX). Before each test, the CPET system underwent a standardized three-step calibration procedure following the manufacturer's specifications: (1) gas analyzer calibration using a certified reference gas mixture (16% O_2_, 5% CO_2_, balance N_2_) and ambient room air; (2) volume/flow calibration of the turbine flowmeter using a 3-L reference syringe across multiple flow rates; and (3) ambient condition correction for barometric pressure, temperature, and humidity. Calibration was repeated if the drift exceeded 1% during the testing day. Cycle ergometer power output was electromagnetically braked and verified monthly against an external reference. As shown in Supplementary Figure 2.

CPET was performed using an individualized ramp protocol. HR, VO_2_, respiratory exchange rate (RER), and ventilation (VE) were collected at rest and AT, respectively. AT was defined by the V-slope method.VE-VCO_2_ slope (VE/VCO_2_) and oxygen uptake efficiency slope (OUES) were calculated on the basis of VO_2_, VE and carbon dioxide output (VCO_2_). Cycling power of AT (WAT) in CPET was also collected to assess participants' exercise performance. Maximum effort was considered to be reached when RER was higher than 1.05. Resting metabolic rate (RMR) Resting metabolic rate was also measured by CPX. Energy expenditure (EE) for RMR was calculated by detecting VO_2_ and VCO_2_ at rest. RMR consists of three components: fat energy (FAT), carbohydrate energy (CHO) and protein energy. Protein energy was set as a constant (405 kcal/day) and the other components were calculated as EE.

All participants received standardized written and verbal instructions ≥24 h before each CPET: to abstain from caffeine, alcohol, and tobacco for at least 12 h; to avoid vigorous exercise for 24 h; to consume a light meal at least 2 h before testing; to wear comfortable athletic attire; and to take all routinely prescribed cardiovascular medications as usual. Upon arrival, each participant received a familiarization briefing on the cycle ergometer, the Borg 6–20 RPE scale, hand signals for symptoms, and the test termination criteria, followed by a 3-min unloaded warm-up and a 1-min seated rest before the incremental ramp protocol began.

The criteria for terminating a CPET follow the AHA guidelines (23): electrocardiogram (ECG) changes indicative of myocardial ischemia and/or severe arrhythmias; abnormal drop in blood pressure (SBP decrease >10 mmHg); excessively high blood pressure (SBP >250 mmHg, DBP >115 mmHg); oxygen saturation ≤80%; RER >1.15; the onset of central nervous system symptoms such as dizziness, ataxia, or confusion; the onset of signs of poor perfusion such as pallor, cyanosis, or cold, clammy skin; or the onset of severe dyspnea. Until termination criteria are met, we use the patient's own exercise tolerance as the indicator for stopping test.

### Statistical analysis

Data was analyzed using R (version 4.4.2) and continuous variables were described by mean and standard deviation. Prior to analysis, raw data were tested for normality using the Shapiro–Wilk test. For normally distributed data, between-group comparisons were made using analysis of variance (ANOVA) and within-group changes from baseline to post-intervention were assessed using t-tests. For non-normally distributed data, non-parametric tests, including the Kruskal–Wallis test, were used. And the statistical results were corrected using false discovery rate (FDR). For the subgroup, conduct a *post-hoc* efficacy analysis. Correlation analyses were performed using. *p* values <0.05 were considered statistically significant. In addition, Pearson's correlation coefficient was used to assess the correlational relationship between exercise and improvement in metabolic outcomes.

## Results

### Participant characteristics

The study included 42 patients with complete data who were eligible for analysis. To explore age-related differences in OUES, participants were divided into two groups based on the mean age: those younger than 55 years (*n* = 22) and those aged 55 years or older (*n* = 20). A summary of their clinical profiles is presented in Table 2. Overall the mean age of the participants was 55 years and 83.33% were male. The cardiovascular disease composition included coronary artery disease in 33 cases (78.57%), valvular disease in 14 cases (33.33%), arrhythmia in 6 cases (14.29%) and unstable angina in 7 cases (16.67%). Additionally, 18 patients (42.86%) had comorbid hypertension, and 9 (21.43%) had diabetes mellitus. More than half of the participants (57.14%) had undergone percutaneous coronary intervention (PCI).

**Table 2:** Baseline characteristics of study participants.

Characteristics	Young group(<55)	Old group(≥55)	All
Sample size	*n* = 22	*n* = 20	*n* = 42
Sex (man)	20 (90.91%)	15 (75%)	35 (83.33%)
CVD	17 (77.27%)	16 (80.00%)	33 (78.57%)
Valve disease	11 (50.00%)	3 (15.00%)	14 (33.33%)
Hypertension	6 (27.27%)	12 (60.00%)	18 (42.86%)
Unstable Angina Pectoris	5 (22.73%)	2 (10.00%)	7 (16.67%)
Arrhythmia	2 (9.09%)	4 (20.00%)	6 (14.29%)
Diabetes	4 (18.18%)	5 (25.00%)	9 (21.43%)
PCI	13 (59.09%)	11 (55.00%)	24 (57.14%)

### Assessment indicators

After one month of rehabilitation training, VO_2_ at the anaerobic threshold (AT) increased significantly, rising from 11.74 ± 3.20 to 13.79 ± 3.48 mL/min/kg (*p* < 0.01). Significant improvements were also observed in ventilation at AT (VE at AT) and the respiratory exchange ratio at AT (RER at AT), increasing from 29.64 ± 7.91 to 35.38 ± 12.22 mL/min/kg (*p* < 0.01) and from 0.92 ± 0.05 to 0.95 ± 0.04 (*p* < 0.01), respectively. Notably, the magnitude of change in VE exceeded that of VO_2_. Among all significantly different variables, VE at AT and VO_2_ at AT showed the highest proportion of change, both exceeding 15%. Additionally, oxygen uptake efficiency slope (OUES) increased significantly from 1,485.20 ± 377.54 to 1,610.60 ± 419.81 (*p* < 0.05). However, no statistically significant differences were found in resting state indicators before and after rehabilitation (*p* > 0.05), as shown in Supplementary Table 1.

**Figure 2:**
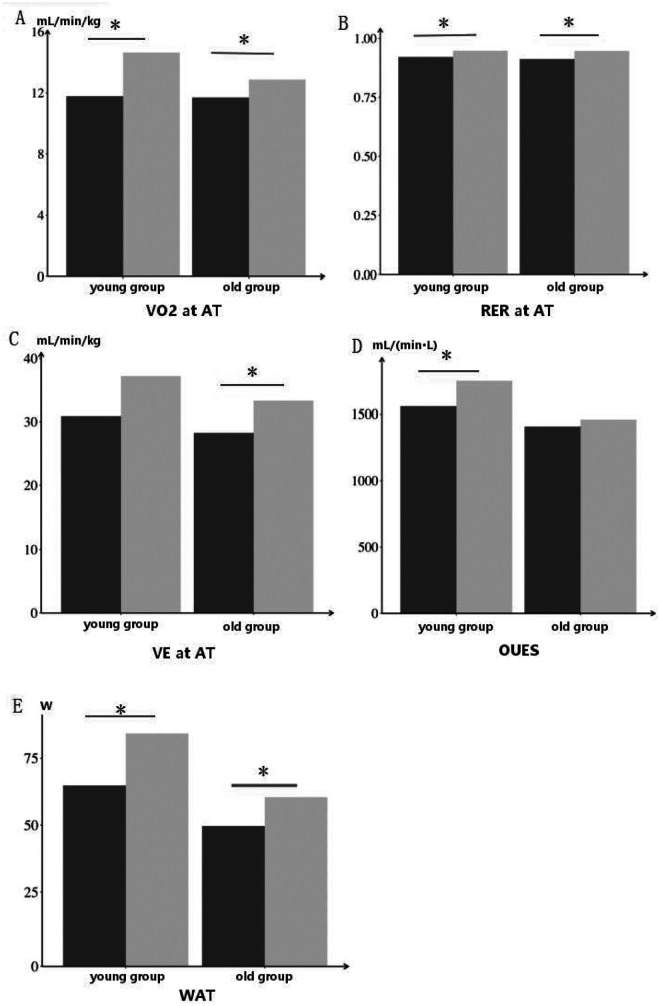
For different age groups, changes in the following indicators before and after training: **(A)** oxygen uptake at reaching the anaerobic threshold; **(B)** respiratory exchange rate at reaching the anaerobic threshold; **(C)** ventilation at reaching the anaerobic threshold; **(D)** slope of oxygen uptake efficiency; **(E)** pedaling power at reaching the anaerobic threshold. * Denotes *P* < 0.05.

VO_2_ at AT, RER at AT, and work at AT (WAT) were significantly higher post-training in both age groups (*p* < 0.05). The changes in VO_2_ at AT and WAT were more pronounced in participants under 55 years of age. While the increase in OUES was statistically significant in the <55-year-old group, it was not significant in the ≥55-year-old group (*p* = 0.49). There were no significant differences in resting heart rate, oxygen uptake, RER, or ventilation before and after training. In contrast, power output at the anaerobic threshold significantly increased regardless of age (*p* < 0.05), as presented in Tables 3, 4 and Figure 2.

**Table 3:** Statistical results of cardiorespiratory indicators in young group.

Variable	Mean ± SD before	Mean ± SD after	Cohen's *d*	95% CI	*t*	*P*	*P* (FDR)
HR at Rest	79.77 ± 14.35	80.23 ± 15.50	−0.04	[−0.46, 0.38]	−0.18	0.8588	0.969
VO2 at Rest	4.12 ± 3.24	4.25 ± 3.50	−0.05	[−0.47, 0.37]	−0.24	0.8126	0.969
RER at Rest	0.79 ± 0.05	0.79 ± 0.05	−0.02	[−0.44, 0.40]	−0.08	0.9370	0.969
VE at Rest	12.50 ± 6.78	12.45 ± 7.32	0.01	[−0.41, 0.43]	0.04	0.9685	0.969
HR at AT	106.64 ± 16.14	111.23 ± 17.43	−0.35	[−0.78, 0.08]	−1.65	0.1138	0.209
VO2 at AT	11.78 ± 5.10	14.64 ± 5.51	−0.75	[−1.23, −0.28]	−3.53	0.0020	0.011*
RER at AT	0.92 ± 0.08	0.95 ± 0.09	−0.64	[−1.09, −0.18]	−2.98	0.0073	0.027*
VE at AT	30.91 ± 18.10	37.23 ± 19.54	−0.43	[−0.87, 0.00]	−2.03	0.0552	0.121
VE/VCO_2_	28.14 ± 5.37	28.06 ± 5.80	0.02	[−0.40, 0.44]	0.11	0.9134	0.969
OUES	1,555.28 ± 462.87	1,746.78 ± 499.90	−0.55	[−1.00, −0.11]	−2.60	0.0165	0.045*
WAT	63.36 ± 27.52	83.32 ± 29.72	−0.81	[−1.30, −0.33]	−3.82	0.0010	0.011*

HR at Rest, quiet state heart rate in beats/min; VO2 at Rest, quiet state oxygen uptake in mL/min/kg; RER at Rest, quiet state respiratory exchange rate; VE at Rest, quiet state ventilation in mL/min/kg; HR at AT, anaerobic threshold heart rate in beats/min; VO2 at AT, anaerobic threshold oxygen uptake in mL/min/kg; RER at AT, anaerobic threshold respiratory exchange rate; VE at AT, anaerobic threshold ventilation in mL/min/kg; VE/VCO2, carbon dioxide ventilation equivalents, VE/VCO_2_ slope; OUES, oxygen uptake efficiency slope in mL/(min·L); WAT, load at anaerobic threshold; *P* (FDR), *P*-values adjusted using false discovery rate.

* Denotes *P* < 0.05.

**Table 4:** Statistical results of cardiorespiratory indicators old groups.

Variable	Mean ± SD before	Mean ± SD after	Cohen's *d*	95% CI	*t*	*P*	*P* (FDR)
HR at Rest	81.10 ± 14.64	75.15 ± 15.81	0.40	[−0.05, 0.85]	1.81	0.0858	0.180
VO2 at Rest	4.99 ± 3.60	4.80 ± 3.89	0.13	[−0.31, 0.57]	0.57	0.5756	0.704
RER at Rest	0.82 ± 0.07	0.86 ± 0.08	−0.39	[−0.84, 0.06]	−1.74	0.0980	0.180
VE at Rest	13.80 ± 4.75	12.60 ± 5.13	0.31	[−0.14, 0.75]	1.38	0.1839	0.289
HR at AT	105.10 ± 19.96	104.45 ± 21.56	0.02	[−0.42, 0.46]	0.11	0.9136	0.914
VO2 at AT	11.70 ± 2.70	12.87 ± 2.92	−0.62	[−1.09, −0.14]	−2.76	0.0124	0.034*
RER at AT	0.91 ± 0.05	0.95 ± 0.06	−0.65	[−1.13, −0.17]	−2.91	0.0089	0.033*
VE at AT	28.25 ± 5.42	33.35 ± 5.86	−0.71	[−1.20, −0.22]	−3.17	0.0050	0.028*
VE/VCO_2_	31.06 ± 4.86	30.92 ± 5.25	0.04	[−0.40, 0.48]	0.17	0.8670	0.914
OUES	1,408.11 ± 272.32	1,460.80 ± 294.11	−0.21	[−0.65, 0.23]	−0.94	0.3592	0.494
WAT	49.70 ± 9.79	59.40 ± 10.57	−0.98	[−1.50, −0.45]	−4.38	0.0003	0.003*

HR at Rest, quiet state heart rate in beats/min; VO2 at Rest, quiet state oxygen uptake in mL/min/kg; RER at Rest, quiet state respiratory exchange rate; VE at Rest, quiet state ventilation in mL/min/kg; HR at AT, anaerobic threshold heart rate in beats/min; VO2 at AT, anaerobic threshold oxygen uptake in mL/min/kg; RER at AT, anaerobic threshold respiratory exchange rate; VE at AT, anaerobic threshold ventilation in mL/min/kg; VE/VCO2, carbon dioxide ventilation equivalents, VE/VCO_2_ slope; OUES, oxygen uptake efficiency slope in mL/(min·L); WAT, load at anaerobic threshold; *P* (FDR), *P*-values adjusted using false discovery rate.

* Denotes *P* < 0.05.

In addition, *post-hoc* subgroup efficacy analysis revealed that the efficacy values for VO_2_ at AT, RER at AT, OUE, and WAT in the younger group, as well as those for VO_2_ at AT, RER at AT, VE at AT, and WAT in the older group, were all greater than 0.50; however, the efficacy values for resting-state indicators and VE/VCO_2_ in both group and OUES in the older group were all less than 0.50, as shown in Supplementary Table 2.

### Correlation analysis

**Figure 3:**
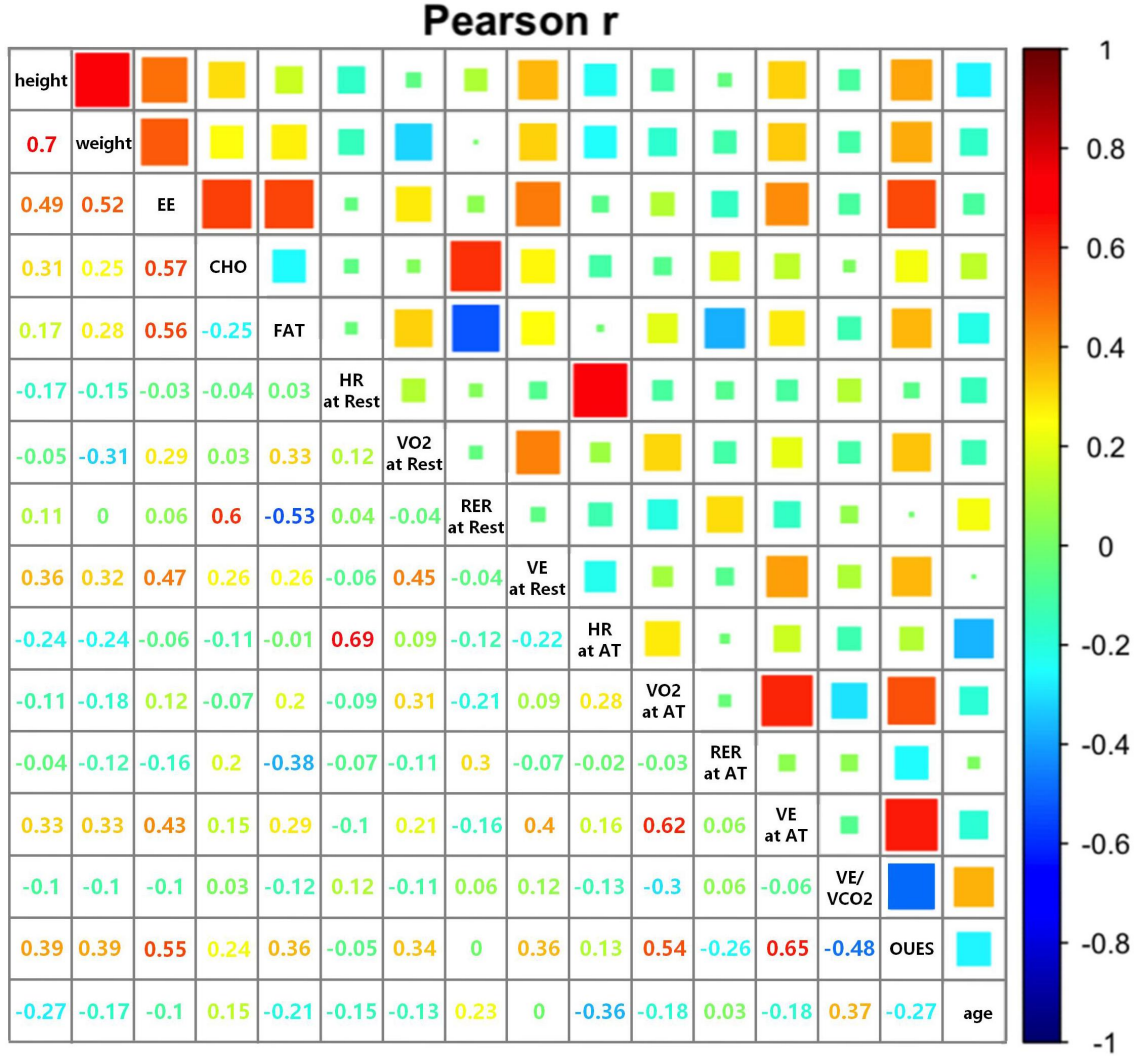
Correlation among indicators: EE, energy provided by substances other than fats and carbohydrates; CHO, energy from carbohydrates; FAT, energy from fat; HR at Rest, quiet state heart rate in beats/min; VO2 at Rest, quiet state oxygen uptake in mL/min/kg; RER at Rest, quiet state respiratory exchange rate; VE at Rest, quiet state ventilation in mL/min/kg; HR at AT, anaerobic threshold heart rate in beats/min; VO2 at AT, anaerobic threshold oxygen uptake in mL/min/kg; RER at AT, anaerobic threshold respiratory exchange rate; VE at AT, anaerobic threshold ventilation in mL/min/kg; VE/VCO2, carbon dioxide ventilation equivalents, VE/VCO_2_ slope; OUES, oxygen uptake efficiency slope in mL/(min·L).

Using Pearson correlation, a pairwise correlation matrix was generated for all valid variables, as illustrated in Figure 3, 4. The analysis revealed correlations between the following pairs: height and body weight; resting heart rate and heart rate at the anaerobic threshold; oxygen uptake at the anaerobic threshold and the oxygen uptake efficiency slope (OUES); and oxygen uptake at the anaerobic threshold with itself, indicating consistency across measurements. Additionally, daily carbohydrate metabolism was correlated with the resting respiratory exchange rate. Total daily energy expenditure showed a moderate correlation with the OUES (*r* = 0.55) and was also correlated with daily carbohydrate and lipid metabolism. However, no significant correlation was observed between total daily metabolism and age.

**Figure 4:**
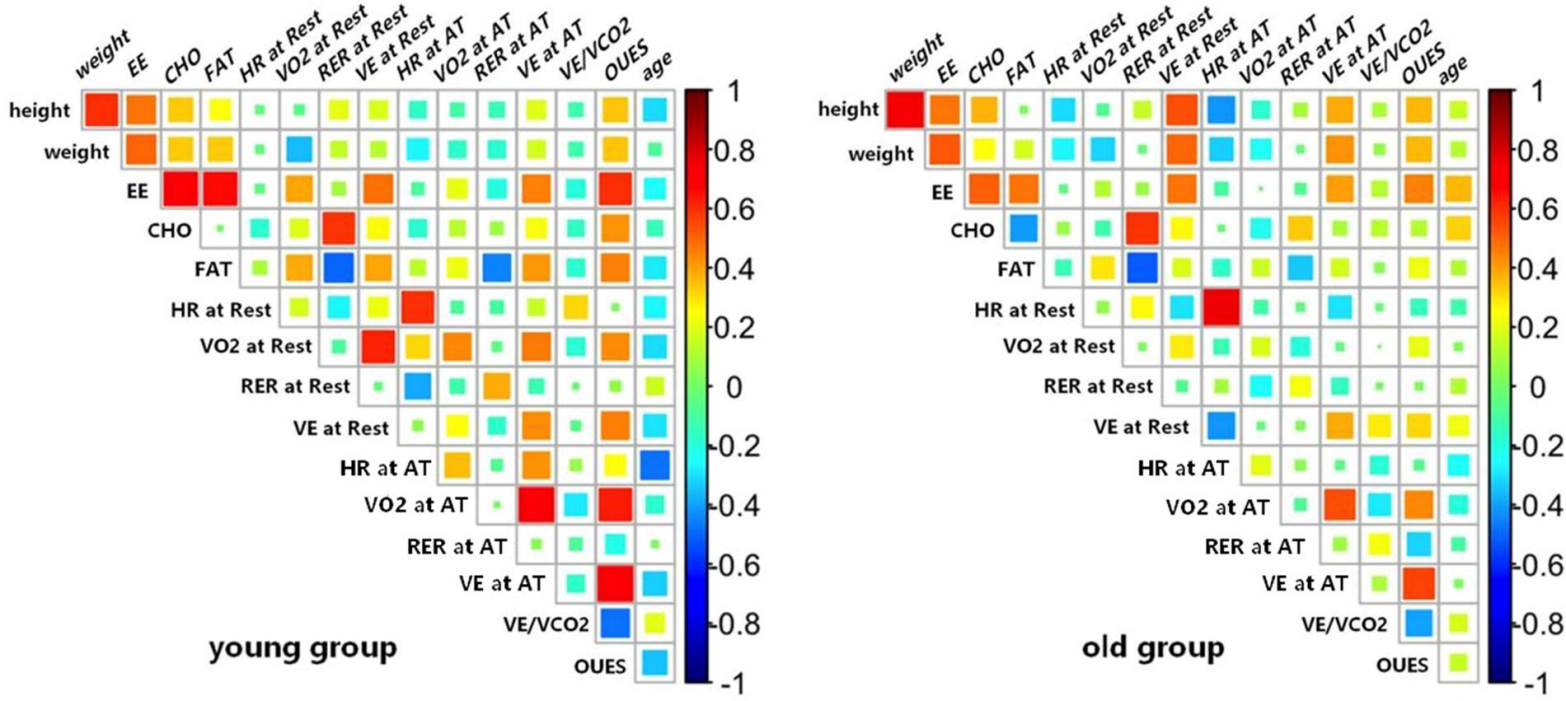
Correlation of indicators across different age groups: EE, total energy consumed per day in kcal; CHO, total energy provided by carbohydrates per day in kcal; FAT, total energy provided by fat per day in kcal; HR at Rest, quiet state heart rate in beats/min; VO2 at Rest, quiet state oxygen uptake in mL/min/kg; RER at Rest, quiet state respiratory exchange rate; VE at Rest, quiet state ventilation in mL/min/kg; HR at AT, anaerobic threshold heart rate in beats/min; VO2 at AT, anaerobic threshold oxygen uptake in mL/min/kg; RER at AT, anaerobic threshold respiratory exchange rate; VE at AT, anaerobic threshold ventilation in mL/min/kg; VE/VCO2, carbon dioxide ventilation equivalents, VE/VCO_2_ slope; OUES, oxygen uptake efficiency slope in mL/(min·L).

## Discussion

The results demonstrated a significant 17.46% improvement in VO_2_ at AT and 26.50% improvement in WAT, with no serious adverse events observed during the program. Based on published data on the natural course of stable cardiovascular disease, it is unlikely that exercise tolerance would spontaneously improve to a degree similar to that observed in this study within a single month (25, 26). Therefore, it can be tentatively concluded that APBCRE has positive rehabilitative effects. The results of the study showed no differences in resting parameters, which may be related to the short duration of the intervention. Current evidence suggests that a one-month cardiac rehabilitation program may not be sufficient to produce statistically significant improvements in resting heart rate and resting oxygen consumption in patients with coronary heart disease. A meta-analysis of patients with coronary heart disease showed that high-intensity interval training (HIIT) and moderate-intensity continuous training (MICT) did not differ significantly in improving resting heart rate and had limited effects on resting oxygen consumption; the improvements were primarily observed in exercise-induced cardiopulmonary function rather than at rest (27). An 8-week study comparing HIIT and continuous exercise training indicated that neither approach resulted in a significant improvement in resting heart rate (28). Additionally, the results of a study on changes in cardiopulmonary fitness in patients with coronary heart disease following a 12-week training program indicated no significant change in resting heart rate before and after training (29). In summary, a short-term rehabilitation program lasting one month may not be sufficient to trigger significant physiological adaptations in resting heart rate and resting oxygen consumption; longer-term, sustained intervention may be required.

The oxygen uptake efficiency slope (OUES) is relatively independent of exercise intensity and correlated with other exercise parameters, making it both discriminatory and sensitive to the effects of physical exercise in cardiac patients. However, OUES values are influenced by anthropometric factors and exhibit considerable inter-individual variability (30). Previous studies have shown that OUES increases similarly following both high-intensity and moderate-intensity interval training (31), suggesting that its improvement may result from both peripheral and central physiological adaptations (32).

Age-related differences observed in this study suggest that OUES and VO_2_ at AT were correlated before and after rehabilitation (*r* = 0.54, *p* < 0.001). Interestingly, OUES demonstrated a significant positive correlation with total energy expenditure (EE) as well (*r* = 0.55, *p* < 0.001). Beyond cardiovascular disease, OUES has also proven effective in assessing cardiorespiratory fitness improvements in patients with chronic stroke, even without increases in VO_2_peak (33). However, under the conditions of this study, OUES showed significant improvement in the <55 age group but did not reach statistical significance in the ≥55 age group, suggesting that age may influence changes in OUES; further research is required. These findings suggest that, in patients under the age of 55, the OUES may serve as a potentially valuable indicator for assessing improvements in cardiopulmonary fitness. Several factors may explain this age-related difference in this unexpected finding. Firstly, older patients exhibit age-related declines in skeletal muscle oxidative capacity (34) and cardiac compliance (35), which may limit the peripheral adaptations that promote improvements in OUES. Second, the proportionate increase in VE at AT was greater than that in VO_2_ at AT in the elderly group, suggesting that the primary benefit for elderly patients may be an improvement in ventilatory efficiency rather than an improvement in oxygen utilisation (36); as OUES integrates these two parameters, a disproportionate ventilatory response may have attenuated the slope. Third, the small sample size in the old group (*n* = 20) may have resulted in insufficient statistical power to detect a small-to-moderate effect (Cohen's *d* = 0.31). Finally, one month of training may have been too short for elderly patients to achieve a measurable improvement in OUES. Therefore, our findings do not support the claim that OUES is age-independent; on the contrary, age appears to alter the responsiveness of this measure to short-term cardiac rehabilitation. Although there is currently no clear literature to support this, it should be noted that EE is calculated based on oxygen consumption and carbon dioxide production (37). We believe that OUES itself is an indicator of oxygen utilization efficiency. To some extent, it reflects the magnitude of oxygen consumption, thereby exhibiting a correlation with EE. This further demonstrates that OUES is a comprehensive indicator with clinical research value; however, the finding of a correlation between OUES and EE will not be included in our conclusions, as further experiments are needed to substantiate it. Consequently, using the OUES as a measure of cardiopulmonary fitness in young patients during exercise rehabilitation minimises the influence of exercise mode and intensity whilst reducing the interference of subjective factors.

This study also has several limitations. First, all participants were recruited from a single center, and the sample had an unbalanced gender distribution. Second, the study used a self-controlled design with no external control group or comparative intervention, which moderately reduced the accuracy and clarity of the findings. Lastly, due to the absence of comparisons between APBCRE and other exercise modalities, this study does not provide direct evidence regarding the evaluative efficacy of OUES across different forms of exercise. The single-arm, non-randomised, non-controlled design constitutes the main limitation of this study. We cannot rule out the possibility that the observed improvements may be partly attributable to spontaneous recovery, regression to the mean, the placebo effect, or natural fluctuations in the disease. In addition, *post-hoc* subgroup analysis revealed that the effect sizes for both resting-state measures and VE/VCO_2_ in both group and OUES in the older group were below 0.50. However, the lack of a significant change may reflect insufficient statistical power or a longer training duration needed for older adults, rather than a genuine age-related non-responsiveness. Consequently, the findings of this study should be regarded as preliminary and hypothesis-generating. A randomised controlled trial with sufficient power, using a control group receiving standard care or exercise, is required to confirm the efficacy of APBCRE. In future research, we aim to conduct multi-center randomized controlled trials, include patients at various stages of disease progression, and extend the intervention period. These improvements will help address current limitations and provide further validation of our findings.

## Summary

Our study suggests that APBCRE has shown preliminary signs of potential efficacy in patients with cardiovascular disease. After one month of APBCRE, patients showed a improvement in exercise tolerance with no serious adverse events observed during the program. Furthermore, the oxygen uptake efficiency slope (OUES) has been shown to be an effective indicator for assessing changes in cardiorespiratory fitness during exercise rehabilitation in people under the age of 55.

### Background

Traditional assessment methods for cardiac rehabilitation is risky and susceptible to subjective factors. Oxygen uptake efficiency slope (OUES) has potential as an alternative as a metric that does not require maximum effort. The purpose of this study was to investigate the clinical effectiveness of adaptive postural balance cardiac rehabilitation exercise (APBCRE) and to validate the effectiveness of OUES in assessing cardiopulmonary rehabilitation outcomes.

### Summary of results

After rehabilitation, patients showed significant increases in anaerobic threshold oxygen uptake, WAT and OUES. Futhermore, OUES was moderately correlated with both anaerobic threshold oxygen uptake and total energy expenditure.

### Potantial significance

APBCRE might be a effective rehabilitation method to rapidly (within 1 month) improve exercise tolerance in patients with CVD, and is particularly suitable for young patients who are unable to complete an extreme test. As a submaximal indicator, OUES may offer a more effective alternative for assessing cardiopulmonary fitness in patients who are unable to perform high-intensity exercise, but its suitability for the elderly remains a matter of debate.

## Data Availability

The raw data supporting the conclusions of this article will be made available by the authors, without undue reservation.
