# Supplementary material for: Alterations in submaximal cardiopulmonary indicators in CVD patients participating in APBCRE—a comprehensive CPET-based study
Source: Front Cardiovasc Med. 2026 Jul 7;13:1825961. doi: 10.3389/fcvm.2026.1825961 (PMC13385174; doi:10.3389/fcvm.2026.1825961)
Supplement: Supplementary file 1 [file Table1.docx]

Supplementary Table 1. Indicators of cardiorespiratory before and after exercise

| **Variable** | **Mean ± SD Before** | **Mean ± SD After** | **Cohen’s d** | **95% CI** | **t** | **P** | **P (FDR)** |
| --- | --- | --- | --- | --- | --- | --- | --- |
| HR at Rest | 80.41 ± 13.19 | 77.81 ± 11.62 | 0.20 | [-0.11 , 0.50] | 1.27 | 0.210 | 0.39 |
| VO2 at Rest | 4.54 ± 1.38 | 4.51 ± 1.56 | 0.06 | [-0.25 , 0.36] | 0.36 | 0.725 | 0.80 |
| RER at Rest | 0.81 ± 0.07 | 0.82 ± 0.08 | -0.19 | [-0.50 , 0.11] | -1.25 | 0.219 | 0.39 |
| VE at Rest | 13.12 ± 3.58 | 12.52 ± 3.83 | 0.13 | [-0.18 , 0.44] | 0.83 | 0.414 | 0.51 |
| HR  at AT | 105.91 ± 16.82 | 108.00 ± 13.25 | -0.17 | [-0.48 , 0.14] | -1.10 | 0.277 | 0.39 |
| VO2 at AT | 11.74 ± 3.20 | 13.79 ± 3.48 | -0.61 | [-0.94 , -0.28] | -3.94 | 0.000 | 0.002** |
| RER at AT | 0.92 ± 0.05 | 0.95 ± 0.04 | -0.53 | [-0.85 , -0.21] | -3.43 | 0.001 | 0.005** |
| VE at AT | 29.64 ± 7.91 | 35.38 ± 12.22 | -0.50 | [-0.82 , -0.18] | -3.25 | 0.002 | 0.006** |
| VE/VCO_2_ | 29.53 ± 4.17 | 29.42 ± 4.76 | 0.03 | [-0.28 , 0.34] | 0.21 | 0.837 | 0.835 |
| OUES | 1485.20 ± 377.54 | 1610.60 ± 419.81 | -0.39 | [-0.70 , -0.07] | -2.51 | 0.016 | 0.035* |
| WAT | 56.86 ± 25.43 | 71.93 ± 26.64 | -0.67 | [-1.01 , -0.33] | -4.35 | 0.000 | 0.0009*** |

HR at Rest: quiet state heart rate in beats/min; VO2 at Rest: quiet state oxygen uptake in ml/min/kg; RER at Rest: quiet state respiratory exchange rate; VE at Rest: quiet state ventilation in ml/min/kg; HR at AT: anaerobic threshold heart rate in beats/min; VO2 at AT: anaerobic threshold oxygen uptake in ml/min/kg; RER at AT: anaerobic threshold respiratory exchange rate; VE at AT: anaerobic threshold ventilation in ml/min/kg; VE/VCO2: carbon dioxide ventilation equivalents, VE/VCO_2_ slope; OUES: oxygen uptake efficiency slope in ml/(min·L); WAT: load at anaerobic threshold; * Denotes P < 0.05; ** Denotes P < 0.01; *** Denotes P < 0.001
