# Supplementary material for: Alterations in submaximal cardiopulmonary indicators in CVD patients participating in APBCRE—a comprehensive CPET-based study
Source: Front Cardiovasc Med. 2026 Jul 7;13:1825961. doi: 10.3389/fcvm.2026.1825961 (PMC13385174; doi:10.3389/fcvm.2026.1825961)
Supplement: Supplementary file 2 [file Table2.docx]

Supplementary Table 2. Post-hoc power analysis for young and old subgroups

| **Variable** | **Young group (n=22) Power** | **Old group (n=20) Power** |
| --- | --- | --- |
| HR at Rest | 0.05 | 0.44 |
| VO2 at Rest | 0.06 | 0.09 |
| RER at Rest | 0.05 | 0.43 |
| VE at Rest | 0.05 | 0.27 |
| HR at AT | 0.25 | 0.05 |
| VO2 at AT | 0.86 | 0.73 |
| RER at AT | 0.69 | 0.76 |
| VE at AT | 0.35 | 0.81 |
| VE/VCO_2_ | 0.05 | 0.05 |
| OUES | 0.58 | 0.17 |
| WAT | 0.90 | 0.95 |

HR at Rest: quiet state heart rate in beats/min; VO2 at Rest: quiet state oxygen uptake in ml/min/kg; RER at Rest: quiet state respiratory exchange rate; VE at Rest: quiet state ventilation in ml/min/kg; HR at AT: anaerobic threshold heart rate in beats/min; VO2 at AT: anaerobic threshold oxygen uptake in ml/min/kg; RER at AT: anaerobic threshold respiratory exchange rate; VE at AT: anaerobic threshold ventilation in ml/min/kg; VE/VCO2: carbon dioxide ventilation equivalents, VE/VCO_2_ slope; OUES: oxygen uptake efficiency slope in ml/(min·L); WAT: load at anaerobic threshold; Power: Power values were calculated by post-hoc analysis for paired t-test using the observed Cohen’s d and subgroup sample sizes.
